# Unveiling a J-shaped association between the triglyceride-glucose index and in-hospital major adverse cardiovascular events in patients with acute myocardial infarction: a retrospective cohort study of 1,065 patients

**DOI:** 10.3389/fcvm.2026.1830398

**Published:** 2026-07-07

**Authors:** Yikang Xu, Jia Liu, Yang Yang, Limin Liu, Jingru Ma, Xiren Meng, Shuochao Zhao, Yaxin Wang, Lisheng Xu, Stephen E. Greenwald

**Affiliations:** 1 Department of Cardiovascular Medicine, The Second Affiliated Hospital of Shenyang Medical College, Shenyang, Liaoning Province, China; 2 Geriatric Medical Center, Shenyang Fourth People’s Hospital, Shenyang, Liaoning Province, China; 3 School of Public Health, Shenyang Medical College, Shenyang, Liaoning Province, China; 4 College of Information Science and Engineering, Northeastern University, Shenyang, Liaoning Province, China; 5 Key Laboratory of Medical Image Computing, Ministry of Education, Shenyang, China; 6 Blizard Institute, Barts & The London School of Medicine & Dentistry, Queen Mary University of London, London, United Kingdom

**Keywords:** acute myocardial infarction, major adverse cardiovascular events, nonlinear association, prognosis, retrospective cohort study, triglyceride-glucose index

## Abstract

**Objective:**

This study was designed to investigate the dose-response relationship between the triglyceride-glucose (TyG) index and the occurrence of in-hospital major adverse cardiovascular events (MACE) in patients with acute myocardial infarction (AMI), and to examine whether a nonlinear association pattern exists.

**Methods:**

This single-center retrospective cohort study consecutively enrolled 1,065 patients diagnosed with type 1 AMI who underwent coronary angiography at the Department of Cardiology, The Second Affiliated Hospital of Shenyang Medical College, between June 2022 and June 2025. Baseline data were collected from the electronic medical record system, and the TyG index was calculated using the formula: TyG = Ln [triglycerides (mg/dL) × fasting plasma glucose (mg/dL)]/2. The primary outcome was in-hospital MACE (occurring during hospitalization, ≤7 days after admission), which was a composite of all-cause mortality, acute heart failure, malignant arrhythmia, recurrent myocardial infarction, and cardiogenic shock. Patients were categorized into three groups [T1 [low], T2 [medium], and T3 [high]] based on the overall tertiles of the TyG index. Multivariable logistic regression analysis was employed to construct hierarchical models (a base model, a parsimonious model, and a core model) and to analyze the association between TyG index groups and MACE. Model performance was evaluated by assessing discrimination (area under the curve, AUC), calibration, and clinical utility. Restricted cubic splines (RCS) were used to explore the potential nonlinear relationship between the TyG index (as a continuous variable) and MACE. The inflection point was identified, and piecewise regression analysis was performed. Subgroup analyses were conducted to assess the heterogeneity of the association.

**Results:**

This study included 1,065 patients with AMI, who were stratified into T1 (low, *n* = 352), T2 (medium, *n* = 361), and T3 (high, *n* = 352) groups based on TyG index tertiles. Baseline analysis revealed significant differences among the three groups in body mass index (BMI), left ventricular ejection fraction (LVEF), age, diastolic blood pressure (DBP), heart rate (HR), fasting plasma glucose (FPG), serum creatinine (Scr), lipid profiles (total cholesterol [TC], triglycerides [TG], high-density lipoprotein cholesterol [HDL-C], low-density lipoprotein cholesterol [LDL-C]), peak cardiac troponin T (cTnT), peak N-terminal pro-B-type natriuretic peptide (NT-proBNP), prevalence of diabetes, and usage rates of angiotensin system inhibitors (ACEI/ARB/ARNI) and beta-blockers (all *p* < 0.05). The overall incidence of in-hospital MACE was 21.13%. Notably, the T2 group exhibited a significantly higher incidence (26.04%) compared to the T1 group (16.76%, *p* = 0.011). In the core multivariable logistic regression model (adjusted for age, sex, BMI, LVEF, diabetes, cTnT, NT-proBNP, etc.), the T2 group was associated with a 178% significantly increased risk of in-hospital MACE compared to the T1 group [odds ratio [OR] = 2.78, 95% confidence interval [CI]: 1.38–5.59, *p* = 0.004]. The core model incorporating the TyG index groups demonstrated the best predictive performance [area under the receiver operating characteristic curve (AUC) = 0.79, 95% CI: 0.75–0.83], good calibration (Hosmer-Lemeshow test *p* = 0.883), and was confirmed to provide clinical net benefit by decision curve analysis. Restricted cubic spline analysis revealed a significant J-shaped relationship between the TyG index and the risk of in-hospital MACE, with an inflection point identified at TyG = 9.05. Piecewise regression analysis demonstrated that when the TyG index was <9.05, the MACE risk did not change significantly (OR = 1.04, 95% CI: 0.62–1.77, *P* = 0.875); however, for every 1-unit increase in the TyG index ≥ 9.05, the MACE risk significantly increased by 124% (OR = 2.24, 95% CI: 1.26–3.96, *P* = 0.006). Subgroup analyses (stratified by sex, smoking status, hypertension, diabetes, age, and BMI) revealed no significant heterogeneity in the association between the TyG index (≥inflection point vs. <inflection point) and MACE risk (all *p* for interaction > 0.05), supporting the consistency of this J-shaped association across the studied population subgroups.

**Conclusion:**

This study demonstrates a significant J-shaped nonlinear association between the TyG index and the risk of in-hospital MACE, with an identified inflection point at 9.05. Notably, patients with a medium TyG level (T2 group) exhibited the highest MACE risk, which was significantly greater than that of patients with a low TyG level (T1 group). This finding challenges the conventional linear perception that a higher TyG index consistently correlates with greater risk. The core model incorporating TyG index stratification significantly improved the predictive performance for MACE. These findings collectively underscore the importance of considering the nonlinear effect of the TyG index in risk assessment for AMI patients, suggesting that individuals with medium TyG levels may represent a specific high-risk subgroup warranting particular attention.

## 1 Introduction

Acute Myocardial Infarction (AMI), a severe manifestation of Coronary Artery Disease (CAD), remains a leading cause of mortality and disability worldwide (1). Despite significant improvements in patient outcomes achieved through Percutaneous Coronary Intervention (PCI) and guideline-directed medical therapy, in-hospital Major Adverse Cardiovascular Events (MACE), including recurrent myocardial infarction, worsening heart failure, malignant arrhythmias, and cardiac death, continue to pose a serious threat to patient lives and substantially increase healthcare burdens (2, 3). Therefore, the early identification of high-risk AMI patients and the implementation of targeted interventions are crucial steps for improving clinical prognosis.

Current risk stratification tools in clinical practice, such as the GRACE score and biomarkers including troponin, natriuretic peptides, and CRP, play a significant role in predicting in-hospital MACE risk in AMI patients (4–6). However, these tools either integrate clinical characteristics or reflect singular pathological processes, such as myocardial injury, hemodynamic stress, or inflammation, thus presenting limitations in assessing underlying metabolic dysregulation. Insulin Resistance (IR), a core pathophysiological cause of metabolic syndrome, is extensively involved in the initiation, progression, and plaque destabilization of atherosclerosis, serving as a critical driver of cardiovascular disease (7). The Triglyceride-glucose (TyG) index, a simple and economical parameter calculated from Fasting Triglycerides (TG) and Fasting Plasma Glucose (FPG) using the expression: TyG = Ln [TG (mg/dL) × FPG (mg/dL)]/2, has been widely validated as a reliable surrogate marker for assessing systemic insulin resistance status (8, 9).

Recent studies have indicated a significant association between the TyG index and various cardiovascular diseases and their prognoses. Multiple investigations have demonstrated a positive correlation between the TyG index and the incidence of MACE, establishing a higher TyG index as an independent predictor of future major adverse cardiovascular events (10, 11). Furthermore, studies in patients with Acute Coronary Syndrome (ACS) have also observed that an elevated TyG index is associated with an increased risk of short-term adverse outcomes (12). However, current studies generally assume a positive linear correlation between the TyG index and cardiovascular risk, and the study populations often encompass the entire ACS spectrum or patients with stable CAD. Research specifically targeting the high-risk AMI subgroup to thoroughly investigate the association between the TyG index and in-hospital MACE remains relatively limited. Notably, evidence regarding a potentially more complex nonlinear relationship (such as a U-shaped or J-shaped association) is particularly scarce. It is noteworthy that some studies suggest that extreme metabolic states (e.g., severely elevated glucose or triglyceride levels) may influence cardiovascular risk through distinct mechanisms, hinting at a potential nonlinear relationship between the TyG index and clinical outcomes (13, 14). Nevertheless, this hypothesis has not been systematically tested in the AMI population.

Therefore, to address this knowledge gap, this study has employed a large-scale retrospective cohort design to thoroughly investigate the nature of the association between the TyG index and the risk of in-hospital MACE in Chinese patients with AMI. It specifically aims to answer the following core scientific questions: (1) Is the TyG index an independent risk factor for in-hospital MACE in Chinese AMI patients? (2) Does a potential nonlinear (specifically J-shaped) relationship exist between them? (3) If a J-shaped relationship is confirmed, does it have an inflection point, and if so, where on the curve does it lie? By addressing these questions, this study seeks to provide novel evidence for refined metabolic risk assessment in AMI patients and to identify potential directions for developing individualized intervention strategies in the future.

## 2 Methods

### 2.1 Study population

This was a single-center, retrospective cohort study. We consecutively enrolled patients who had undergone coronary angiography and were diagnosed with Acute Myocardial Infarction (AMI) [specifically, type 1 MI according to the Fourth Universal Definition of Myocardial Infarction (2018) (15)] at the Department of Cardiology, The Second Affiliated Hospital of Shenyang Medical College, between June 2022 and June 2025. The exclusion criteria were as follows: AMI caused by non-atherosclerotic etiologies (e.g., coronary artery spasm, embolism, dissection, or myocarditis); concomitant severe hepatic failure (Child-Pugh class C), severe renal failure [estimated glomerular filtration rate (eGFR) < 15 mL/min/1.73 m^2^ or requiring dialysis], coagulation disorders, cardiomyopathy, moderate-to-severe valvular heart disease, respiratory failure, active severe infection, uncontrolled thyroid disease, active hematological diseases, autoimmune diseases, or malignant tumors; missing key baseline clinical or laboratory data; and death before completing necessary baseline examinations. Additionally, patients aged <18 years were excluded. A total of 1,065 eligible patients were ultimately included. The study was approved by the Ethics Committee of The Second Affiliated Hospital of Shenyang Medical College (Approval No.: 2025-SYEY-EC-30). All patients provided routine informed consent for coronary angiography. The Ethics Committee waived the requirement for specific research-informed consent due to the study's retrospective nature and the use of anonymized data only.

### 2.2 Data collection

Baseline data were retrospectively collected from the electronic medical record system, encompassing the following categories:

1. **Demographics and Medical History:** age, sex, body mass index (BMI), smoking history (defined as having smoked at least one cigarette per day for ≥6 months), alcohol consumption history (defined as consuming alcohol at least once per week with an equivalent alcohol intake ≥50 mL for ≥6 months), history of hypertension (systolic blood pressure ≥140 mmHg and/or diastolic blood pressure ≥90 mmHg, or previously diagnosed and receiving antihypertensive medication), history of diabetes mellitus (diagnosed according to WHO criteria or under active treatment), family history of coronary heart disease, history of atrial fibrillation (AF), history of peripheral artery disease (PAD), history of chronic kidney disease (CKD), and history of stroke.
2. **Vital Signs on Admission:** systolic blood pressure (SBP), diastolic blood pressure (DBP), mean arterial pressure (MAP), and heart rate (HR). All vital signs were measured at rest in the supine position within the first 10 min after emergency department arrival. SBP and DBP were obtained using a standard automated oscillometric device (brachial cuff). MAP was calculated as: MAP = DBP + 1/3(SBP − DBP). HR was recorded via simultaneous electrocardiogram monitoring or palpation of the radial pulse over 30 s.
3. **Blood Chemistry:** total cholesterol (TC), triglycerides (TG), high-density lipoprotein cholesterol (HDL-C), low-density lipoprotein cholesterol (LDL-C), peak cardiac troponin T (cTnT), peak creatine kinase-MB (CK-MB), peak N-terminal pro-B-type natriuretic peptide (NT-proBNP), fasting plasma glucose (FPG), serum creatinine (Scr), uric acid (UA), and C-reactive protein (CRP). The triglyceride-glucose (TyG) index was calculated using the formula: TyG = Ln [TG (mg/dL) × FPG (mg/dL)]/2.
4. **Cardiac Structure and Function:** left ventricular ejection fraction (LVEF) and left ventricular end-diastolic dimension (LVEDD). These parameters were assessed using transthoracic echocardiography (TTE) performed within 24–48 h after admission, in accordance with routine clinical practice at our center. Echocardiographic examinations were conducted by experienced sonographers using standardized imaging protocols on available ultrasound systems (Philips EPIQ CVx). LVEF was measured using the biplane Simpson's method from the apical four- and two-chamber views. LVEDD was obtained from the parasternal long-axis view using M-mode or 2D-guided measurements at end-diastole. All measurements were performed in accordance with current American Society of Echocardiography/European Association of Cardiovascular Imaging recommendations and were reviewed by a senior cardiologist for consistency.
5. **Coronary Angiography:** All patients underwent selective coronary angiography via the radial or femoral approach. The degree of stenosis in the major coronary arteries (left main [LM], left anterior descending [LAD], left circumflex [LCX], right coronary artery [RCA], and their major primary branches [e.g., diagonal branches, obtuse marginal branches]) was independently assessed by two experienced interventional cardiologists using quantitative coronary angiography (QCA) software (Siemens QAngio XA version 7.3 or equivalent, with an automated edge-detection algorithm) in a blinded fashion. Prior to analysis, the catheter tip was used for automated calibration. The minimal lumen diameter (MLD) and reference vessel diameter were measured in the projection that showed the most severe stenosis, typically at end-diastole. Percent diameter stenosis was automatically calculated as: (1 − MLD/reference diameter) × 100%. The interobserver agreement for the measurement of percent diameter stenosis was assessed using the intraclass correlation coefficient (ICC), which showed excellent consistency (ICC = 0.92, 95% CI: 0.89–0.94). In cases of discrepancy (>10% difference in stenosis quantification or disagreement on lesion significance), a consensus was reached through discussion, and if needed, adjudicated by a third senior interventional cardiologist. Multivessel coronary artery disease (MVD) was defined as the presence of ≥50% diameter stenosis in ≥two major coronary arteries or their major primary branches, or the presence of left main disease.
6. **Treatments:** Use of angiotensin-converting enzyme inhibitors/angiotensin receptor blockers/angiotensin receptor-neprilysin inhibitors (ACEI/ARB/ARNI) or beta-blockers (*β*-blockers), and the number of stents implanted during percutaneous coronary intervention (PCI).
7. **Primary Outcome—In-hospital Major Adverse Cardiovascular Events (MACE)** (occurring during hospitalization, ≤7 days): a composite endpoint comprising all-cause death, acute heart failure (new onset or worsening symptoms/signs of heart failure accompanied by elevated natriuretic peptide levels), malignant arrhythmia (severe sinus bradycardia ≤40 beats/min, second-degree or higher atrioventricular block, ventricular tachycardia, ventricular fibrillation, or cardiac arrest), recurrent myocardial infarction (typical ischemic symptoms with dynamic electrocardiographic changes and troponin levels meeting reinfarction criteria), and cardiogenic shock (systolic blood pressure < 90 mmHg for >30 min or requiring vasoactive drug support, accompanied by evidence of tissue hypoperfusion).

### 2.3 Statistical analysis

Patients were divided into three groups according to their TyG tertile (derived from the entire study cohort, *n* = 1,065): T1 (low TyG), T2 (medium TyG), and T3 (high TyG). Patients were stratified into three groups (tertiles) based on their TyG index: T1 (low TyG: <8.60), T2 (medium TyG: 8.60–9.29), and T3 (high TyG: ≥9.30), each comprising approximately one-third of the cohort. Continuous variables were tested for normality using the Shapiro–Wilk test. Normally distributed data are presented as mean ± standard deviation (SD) and were compared among groups using one-way analysis of variance (ANOVA). Non-normally distributed data are presented as median [interquartile range (IQR)] and were compared using the Kruskal–Wallis test, with *post-hoc* pairwise comparisons adjusted by the Bonferroni correction. Categorical variables are expressed as frequency (percentage) [n (%)] and were compared using the Chi-square test or Fisher's exact test, as appropriate. For the head-to-head comparison between the T2 and T3 TyG tertiles, we used the same statistical framework as the overall tertile group comparison, with additional adjustments for multiple testing. Categorical variables were compared with the Pearson *χ*^2^ test (or Fisher's exact test for small cell counts), and continuous variables were compared with the independent samples t-test (normal distribution) or Mann–Whitney U test (non-normal distribution).

The association between the TyG index and in-hospital MACE was assessed using multivariable logistic regression analysis, with hierarchical models constructed as follows: Model 1 (Base Model) forced the inclusion of clinically relevant variables such as age, sex, BMI, LVEF, and diabetes mellitus; Model 2 (Parsimonious Model) retained significant variables (*p* < 0.05) in the univariate analysis; Model 3 (Core Model) added TyG index groups (using T1 as the reference) to Model 2. A nomogram was developed based on the final model to visualize individual risk. Model performance was evaluated in three ways: (1) Discrimination, assessed by the area under the receiver operating characteristic curve (AUC) and its 95% confidence interval (CI); (2) Calibration, evaluated using calibration curves and the Hosmer-Lemeshow test (a *p*-value > 0.05 indicating good fit); and (3) Clinical utility, determined by decision curve analysis (DCA) to estimate the net benefit. To further evaluate the incremental predictive value and clinical utility of TyG stratification beyond existing predictors, we compared Model 3, which included TyG tertile grouping, with Model 2, which did not include TyG. Model 2 incorporated variables that were statistically significant in the univariate logistic regression analysis, excluding TyG. The AUC and DeLong test were used to compare the models' discriminatory power; We used Categorical NRI, Continuous NRI, and IDI to evaluate risk reclassification and discriminatory improvement. The risk stratification for Categorical NRI was preset at <10%, 10%–20%, 20%–30%, and ≥30%. We re-performed decision curve analysis to compare four strategies: treat-none, treat-all, Model 2 (without TyG), and Model 3 (with TyG). The clinically actionable intervention was defined as escalating to intensive in-hospital monitoring for patients predicted to be at high risk. Given that the overall in-hospital MACE incidence at our institution is approximately 21.7%, 20% was preset as the primary clinically relevant threshold.

Restricted cubic splines (RCS) with 4 knots were employed to explore the potential nonlinear relationship between the continuous TyG index and MACE. The likelihood ratio test was used to confirm the significance of the nonlinear association (*p* for nonlinearity < 0.05). Upon identifying a significant nonlinear relationship, an inflection point was identified, and piecewise logistic regression analysis was performed on either side of this point. Subgroup analyses were conducted, stratifying by sex, smoking status, hypertension, diabetes, age (<65 years/≥65 years), and BMI (<30 kg/m^2^/ ≥ 30 kg/m^2^). The association between the TyG index (categorized as ≥inflection point vs. < inflection point) and MACE risk was examined within each subgroup, and interaction effects were tested using an interaction term (a *p* value for interaction < 0.05 was considered statistically significant for heterogeneity).

All statistical analyses were performed using R software (version 4.3.2). Multicollinearity was assessed using the variance inflation factor (VIF), with a VIF < 5 considered acceptable. Missing data, which accounted for less than 5% of the total, were handled using multiple imputation by chained equations (MICE). The variables with missing data included uric acid (UA), C-reactive protein (CRP), creatine kinase-MB (CK-MB), smoking history, and drinking history. A two-sided *p*-value < 0.05 was considered statistically significant for all tests.

#### 2.3.1 Interaction analysis

To formally evaluate whether the prognostic impact of the TyG index on in-hospital MACE was modified by the use of key cardiovascular medications, multiplicative interaction analyses were performed. In the fully adjusted core multivariable logistic regression model (Model 3), two-way interaction terms between the TyG index tertile groups (T1, T2, T3) and the use of ACEI/ARB/ARNI or beta-blockers (both modeled as binary variables) were separately introduced. Statistical significance of the interaction was assessed using the Wald test for the cross-product term, with a two-sided *p*-value for interaction <  0.05 considered indicative of a significant effect modification. To illustrate the patterns, stratified odds ratios (OR) with 95% confidence intervals (CIs) for the association between TyG tertiles and MACE were calculated within the subgroups of users and non-users for each medication class.

### 2.4 Sensitivity analysis

To mitigate the concern of reverse causality, wherein cardiogenic shock at admission might acutely distort the triglyceride-glucose (TyG) index, a pre-specified sensitivity analysis was performed. All patients who presented with cardiogenic shock on admission (*n* = 32) were excluded, and the core analyses were repeated in the remaining 1,033 patients. Specifically, the nonlinear association was re-evaluated using restricted cubic splines, and the independent predictive value of the TyG index tertiles for in-hospital MACE was reassessed in the fully adjusted Core Model (Model 3).

## 3 Results

### 3.1.1 Baseline characteristics of the study population

A total of 1,065 eligible patients with AMI were ultimately included in this study. Based on the overall tertiles of the TyG index, patients were stratified into three groups: T1 (low TyG, *n* = 352, 33.05%), T2 (medium TyG, *n* = 361, 33.90%), and T3 (high TyG, *n* = 352, 33.05%). The comparisons of baseline characteristics among the three groups are detailed in Table 1.

**Table 1:** Baseline characteristics of the study population according to TyG tertiles.

Variables	Total (*n* = 1,065)	TyG tertile1 (*n* = 352)	TyG tertile2 (*n* = 361)	TyG tertile3 (*n* = 352)	Statistic	*P* value
Demographic characteristics						
Age, years	62.75 ± 12.36	64.64 ± 11.58	64.28 ± 12.37	59.30 ± 12.41	F = 21.39	<0.001
Sex, *n* (%)					*χ*^2^ = 4.40	0.111
Male	820 (77.0)	284 (80.7)	268 (74.2)	268 (76.1)		
Female	245 (23.0)	68 (19.3)	93 (25.8)	84 (23.9)		
Body mass index, kg/m^2^	25.66 ± 3.89	24.34 ± 3.50	25.13 ± 3.53	27.30 ± 3.95	F = 41.33	<0.001
Vital signs and cardiac function						
Heart rate, bpm	76.37 ± 16.98	75.49 ± 17.95	74.75 ± 16.52	78.89 ± 16.20	F = 6.03	0.002
Systolic blood pressure, mmHg	135.48 ± 24.92	134.14 ± 25.08	136.46 ± 25.52	135.81 ± 24.15	F = 0.82	0.442
Diastolic blood pressure, mmHg	81.50 ± 15.31	79.76 ± 15.15	82.16 ± 15.52	82.58 ± 15.16	F = 3.50	0.031
Mean arterial pressure, mmHg	99.40 ± 17.70	97.89 ± 17.30	99.98 ± 18.48	100.33 ± 17.21	F = 1.97	0.14
Left ventricular ejection fraction, %	55.11 ± 8.70	56.38 ± 8.39	54.56 ± 8.46	54.41 ± 9.13	F = 5.66	0.004
Left ventricular diameter, mm	49.90 ± 6.23	49.81 ± 6.19	49.70 ± 6.30	50.19 ± 6.18	F = 0.53	0.587
Laboratory biomarkers						
Fasting plasma glucose, mmol/L	7.85 ± 3.87	6.03 ± 1.85	6.88 ± 2.29	10.22 ± 4.96	F = 135.44	<0.001
Triglyceride–glucose (TyG) index	8.96 (8.34–9.49)	8.03 (7.37–8.33)	8.96 (8.80–9.10)	9.78 (9.50–10.17)	χ^2^ = 946.69	<0.001
Total cholesterol, mmol/L	4.51 ± 1.18	4.15 ± 1.04	4.47 ± 1.08	4.89 ± 1.27	F = 35.95	<0.001
Triglycerides, mmol/L	1.91 ± 1.49	1.08 ± 0.71	1.49 ± 0.40	3.04 ± 1.96	F = 221.73	<0.001
HDL-C, mmol/L	1.09 ± 0.29	1.17 ± 0.31	1.09 ± 0.27	1.00 ± 0.28	F = 30.47	<0.001
LDL-C, mmol/L	2.86 ± 0.94	2.59 ± 0.85	2.92 ± 0.93	3.05 ± 0.98	F = 22.98	<0.001
Serum creatinine, μmol/L	81.31 ± 36.54	80.93 ± 32.74	85.67 ± 45.85	77.12 ± 27.66	F = 4.76	0.009
Uric acid, μmol/L	355.75 ± 144.67	340.25 ± 104.46	365.32 ± 192.35	360.50 ± 117.54	F = 2.45	0.087
Cardiac troponin T, pg/mL	3.63 (0.75–12.41)	2.30 (0.56–11.29)	3.65 (1.00–12.00)	4.40 (0.89–13.09)	χ^2^ = 6.70	0.035
N-terminal pro-B-type natriuretic peptide, pg/mL	1,100.00 (399.10–2,422.50)	886.50 (355.77–1,840.72)	1,145.00 (466.38–2,053.95)	1,359.00 (406.10–4,750.75)	χ^2^ = 16.29	<0.001
C-reactive protein, mg/L	10.15 (3.96–27.41)	10.60 (3.20–28.61)	10.00 (3.24–24.20)	10.04 (5.00–30.08)	χ^2^ = 2.13	0.344
Creatine kinase-MB, U/L	30.00 (7.90–135.97)	24.38 (5.41–118.00)	33.05 (7.69–140.00)	35.90 (10.00–139.37)	χ^2^ = 4.01	0.135
Clinical history and treatments						
Hypertension, *n* (%)	615 (57.8)	187 (53.3)	210 (58.2)	218 (61.9)	χ^2^ = 5.43	0.066
Diabetes mellitus, *n* (%)	376 (35.4)	75 (21.5)	110 (30.5)	191 (54.3)	χ^2^ = 88.12	<0.001
Smoking, *n* (%)	509 (48.1)	165 (47.6)	176 (48.8)	168 (47.9)	χ^2^ = 0.11	0.946
Drinking, *n* (%)	322 (30.6)	100 (29.2)	104 (29.0)	118 (33.6)	χ^2^ = 2.25	0.324
Atrial fibrillation, *n* (%)	80 (7.6)	35 (10.1)	21 (5.8)	24 (6.9)	χ^2^ = 5.01	0.082
Stroke, *n* (%)	162 (15.3)	51 (14.7)	63 (17.5)	48 (13.6)	χ^2^ = 2.16	0.34
Peripheral artery disease, *n* (%)	47 (4.5)	19 (5.5)	20 (5.6)	8 (2.3)	χ^2^ = 5.85	0.054
Chronic kidney disease, *n* (%)	43 (4.1)	15 (4.4)	18 (5.1)	10 (2.9)	χ^2^ = 2.23	0.328
Multivessel disease, *n* (%)	801 (78.8)	260 (79.5)	262 (75.3)	279 (81.8)	χ^2^ = 4.53	0.104
ACEI/ARB/ARNI use, *n* (%)	565 (53.5)	169 (48.7)	182 (50.8)	214 (61.0)	χ^2^ = 12.10	0.002
*β*-blocker use, *n* (%)	706 (66.9)	216 (62.3)	225 (62.9)	265 (75.5)	χ^2^ = 17.75	<0.001
Clinical outcome						
In-hospital MACE, *n* (%)	230 (21.7)	59 (16.8)	95 (26.3)	77 (21.9)	χ^2^ = 9.59	0.008
All-cause death *n* (%)	18 (1.7)	9 (2.6)	7 (1.9)	2 (0.6)	χ^2^ = 4.39	0.111
Acute heart failure *n* (%)	62 (5.8)	24 (6.8)	25 (6.9)	13 (3.7)	χ^2^ = 4.35	0.114
Malignant arrhythmia *n* (%)	135 (12.6)	35 (9.9)	49 (13.6)	51 (14.5)	χ^2^ = 3.68	0.159
Recurrent myocardial infarction *n* (%)	19 (1.8)	4 (1.1)	12 (3.3)	3 (0.9)	χ^2^ = 7.47	0.024
Cardiogenic shock *n* (%)	32(3.0)	13(3.7)	12(3.3)	7(2.0)	χ^2^ = 1.95	0.378

Values are presented as mean ± standard deviation, median (interquartile range), or number (percentage). Differences among groups were assessed using one-way ANOVA or Kruskal–Wallis test for continuous variables and chi-square test for categorical variables, as appropriate.

TyG, triglycerid–glucose index; HDL-C, high-density lipoprotein cholesterol; LDL-C, low-density lipoprotein cholesterol; NT-proBNP, N-terminal pro-B-type natriuretic peptide; MACE, major adverse cardiovascular events.

The results show that Body Mass Index (BMI), Left Ventricular Ejection Fraction (LVEF), age, Diastolic Blood Pressure (DBP), Heart Rate (HR), Fasting Plasma Glucose (FPG), Serum Creatinine (Scr), Total Cholesterol (TC), Triglycerides (TG), High-Density Lipoprotein Cholesterol (HDL-C), Low-Density Lipoprotein Cholesterol (LDL-C), peak cardiac Troponin T (cTnT), peak N-terminal pro-B-type Natriuretic Peptide (NT-proBNP), prevalence of diabetes mellitus, and the utilization rates of angiotensin system inhibitors (ACEI/ARB/ARNI) and beta-blockers differed significantly among the three groups (all *p* < 0.05).

Regarding clinical outcomes, the overall incidence of in-hospital MACE was 21.7%. The univariate analysis revealed a statistically significant difference in MACE incidence among the three TyG index groups (16.8% in T1 vs. 26.3% in T2 vs. 21.9% in T3; *χ*^2^ = 9.59, *p* = 0.008). Subsequent pairwise comparisons (without adjustment for multiple testing) indicated that the risk in the T2 group was significantly higher than that in the T1 group (*p* = 0.003), and the incidence in T2 was also numerically higher than that in T3. The incidence of individual MACE components across TyG index tertiles is presented in Table 1. Malignant arrhythmia was the most frequent MACE component across all groups, with an incidence of 9.9% in the low TyG group (T1), 13.6% in the medium TyG group (T2), and 14.5% in the high TyG group (T3). Recurrent myocardial infarction was the only component with a statistically significant difference across the three groups (*P* = 0.024), with the highest incidence observed in the T2 group (3.3%). No significant differences were observed in the incidence of all-cause death, acute heart failure, or cardiogenic shock across the TyG tertiles (all *p* > 0.05). No statistically significant differences were observed among the three groups for the remaining clinical indicators (all *p* > 0.05), including cardiac structure (LVEDD), vital signs (SBP, MAP), blood chemistry (UA, CRP, CK-MB), as well as demographics and comorbidities (sex, smoking, alcohol consumption, hypertension, family history of CHD, AF, PAD, CKD, stroke, MVD) and number of stents implanted.

### 3.1.2 Head-to-head comparison between the T2 and T3 groups

To examine whether the counterintuitive T2 risk excess was driven by an imbalance in conventional clinical prognosticators, we performed a direct comparison between the T2 and T3 groups (Table 2). Although T2 patients were significantly older (64.28 ± 12.37 vs. 59.30 ± 12.41 years, *p* < 0.001), the groups did not differ in established surrogates of infarct severity (peak cTnT, CK-MB, NT-proBNP), cardiac structure and function (LVEF, LVEDD), or the prevalence of multivessel coronary artery disease (75.3% vs. 81.8%, *p* = 0.051) and admission cardiogenic shock (3.3% vs. 2.0%, *p* = 0.650) (all *p* > 0.05 for these comparisons). These findings argue against the explanation that the elevated MACE risk in the T2 group reflects more extensive myocardial damage, worse cardiac function, or greater hemodynamic compromise relative to the T3 group.

**Table 2:** Comparison of Key clinical characteristics between T2 and T3 groups.

Variables	TyG tertile2 (*n* = 361)	TyG tertile3 (*n* = 352)	Statistic	*P* value
Age, years	64.28 ± 12.37	59.30 ± 12.41	F = 28.912	<0.001
Left ventricular ejection fraction, %	54.56 ± 8.46	54.41 ± 9.13	F = 0.024	0.875
Left ventricular diameter, mm	49.70 ± 6.30	50.19 ± 6.18	F = 1.173	0.241
Cardiac troponin T, pg/mL	3.30 (0.81–10.99)	3.07 (0.56–10.00)	χ^2^ = 1.164	0.245
N-terminal pro-B-type natriuretic peptide, pg/mL	974.69 (352.00–2,053.95)	841.79 (315.00–1,880.00)	χ^2^ = 0.856	0.329
Creatine kinase-MB, U/L	33.30 (7.83–139.75)	34.00 (9.26–140.74)	χ^2^ = −0.696	0.487
Multivessel disease, *n* (%)	262 (75.3)	279 (81.8)	χ^2^ = 0.408	0.051
Cardiogenic shock *n* (%)	12 (3.3)	7 (2.0)	χ^2^ = 0.21	0.650

### 3.2 Multi-model analysis of the association between the TyG index and MACE

#### 3.2.1 Univariable logistic regression analysis

Results from the univariable analysis (Table 3) revealed that the TyG index, as a continuous variable, was significantly and positively associated with the risk of in-hospital MACE [Odds Ratio [OR] = 1.38, 95% Confidence Interval [CI]: 1.21–1.59, *p* < 0.001], indicating a 38% increase in MACE risk per 1-unit increment in the TyG index. When analyzed by TyG index tertiles, the T2 group (medium TyG) had a significantly higher risk of MACE compared to the T1 group (low TyG) (OR = 1.75, 95% CI: 1.21–2.52, *P* = 0.003). Other factors significantly associated with an increased risk of MACE included: diabetes mellitus (DM, OR = 1.38), atrial fibrillation (AF, OR = 3.99), chronic kidney disease (CKD, OR = 2.23), BMI (OR = 1.05), age (OR = 1.03), heart rate (HR, OR = 1.04), serum creatinine (Scr, OR = 1.01), peak cardiac troponin T (cTnT, OR = 1.02), peak NT-proBNP (OR = 1.01), and fasting plasma glucose (FPG, OR = 1.07) (all *p* < 0.05). Factors significantly associated with a decreased risk of MACE included the use of ACEI/ARB/ARNI medications (OR = 0.68) and left ventricular ejection fraction (LVEF, OR = 0.96) (all *p* < 0.05).

**Table 3:** Univariate logistic regression analysis for in-hospital MACE.

Variables	β	S.E.	Z value	*P* value	OR (95% CI)
Triglyceride–glucose tertiles					
Tertile 1					1.00 (Reference)
Tertile 2	0.56	0.19	3	0.003	1.75 (1.21–2.52)
Tertile 3	0.33	0.19	1.71	0.086	1.39 (0.95–2.03)
Sex					
Female					1.00 (Reference)
Male	−0.19	0.17	−1.08	0.282	0.83 (0.59–1.16)
Smoking					
No					1.00 (Reference)
Yes	0.04	0.15	0.29	0.773	1.04 (0.78–1.40)
Drinking					
No					1.00 (Reference)
Yes	−0.02	0.16	−0.13	0.898	0.98 (0.71–1.35)
Hypertension					
No					1.00 (Reference)
Yes	−0.04	0.15	−0.29	0.77	0.96 (0.71–1.29)
Diabetes mellitus					
No					1.00 (Reference)
Yes	0.32	0.15	2.11	0.035	1.38 (1.02–1.86)
Atrial fibrillation					
No					1.00 (Reference)
Yes	1.38	0.24	5.81	<0.001	3.99 (2.50–6.36)
Chronic kidney disease					
No					1.00 (Reference)
Yes	0.8	0.32	2.47	0.013	2.23 (1.18–4.22)
Stroke					
No					1.00 (Reference)
Yes	0.2	0.2	1.01	0.312	1.22 (0.83–1.81)
Multivessel disease					
No					1.00 (Reference)
Yes	−0.23	0.18	−1.28	0.199	0.79 (0.56–1.13)
ACEI/ARB/ARNI use					
No					1.00 (Reference)
Yes	−0.38	0.15	−2.54	0.011	0.68 (0.51–0.92)
β-blocker use					
No					1.00 (Reference)
Yes	−0.10	0.16	−0.60	0.548	0.91 (0.67–1.24)
Body mass index, kg/m^2^	0.05	0.02	2.1	0.035	1.05 (1.01–1.10)
Cardiac troponin T, pg/mL	0.02	0	6.33	<0.001	1.02 (1.02–1.03)
NT-proBNP, pg/mL	0.01	0	8.05	<0.001	1.01 (1.01–1.01)
Left ventricular ejection fraction, %	−0.04	0.01	−5.54	<0.001	0.96 (0.94–0.97)
Age, years	0.03	0.01	5.09	<0.001	1.03 (1.02–1.05)
Left ventricular diameter, mm	0.04	0.01	3.01	0.003	1.04 (1.01–1.06)
Fasting plasma glucose, mmol/L	0.07	0.02	3.56	<0.001	1.07 (1.03–1.11)
Serum creatinine, μmol/L	0.01	0	2.84	0.004	1.01 (1.01–1.01)
Triglyceride–glucose index	0.32	0.07	4.62	<0.001	1.38 (1.21–1.59)

Odds ratios (ORs) and 95% confidence intervals (CIs) were estimated using univariate logistic regression analysis.

OR, odds ratio; CI, confidence interval; TyG, triglyceride–glucose index; NT-proBNP, N-terminal pro-B-type natriuretic peptide; MACE, major adverse cardiovascular events.

#### 3.2.2 Multivariable logistic regression analysis

**Figure 1:**
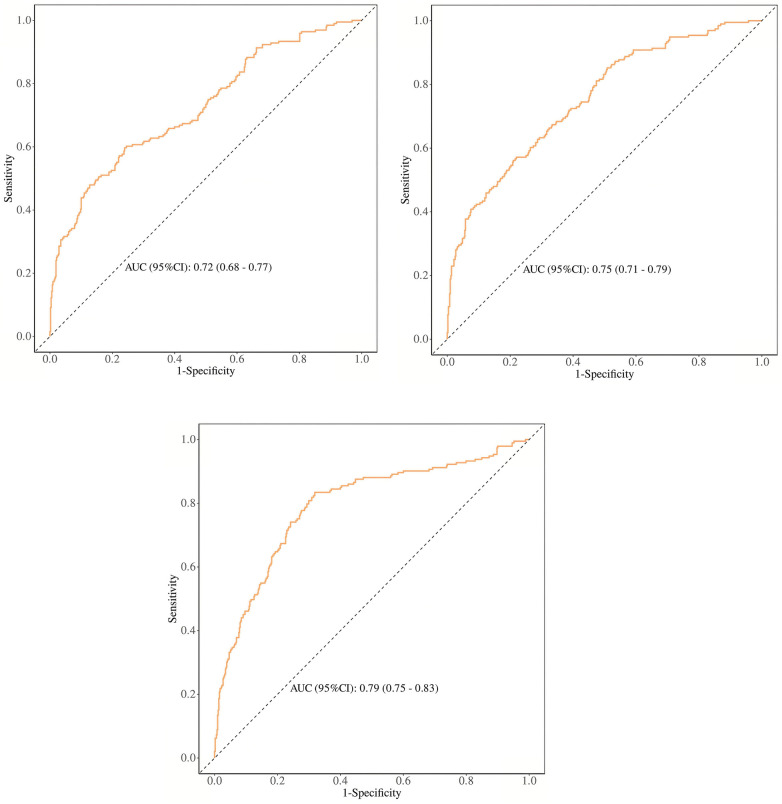
Area under the ROC for each model. **(a)** Model 1 (Base Model). **(b)** Model 2 (Parsimonious Model). **(c)** Model 3 (Core Model).

**Figure 2:**
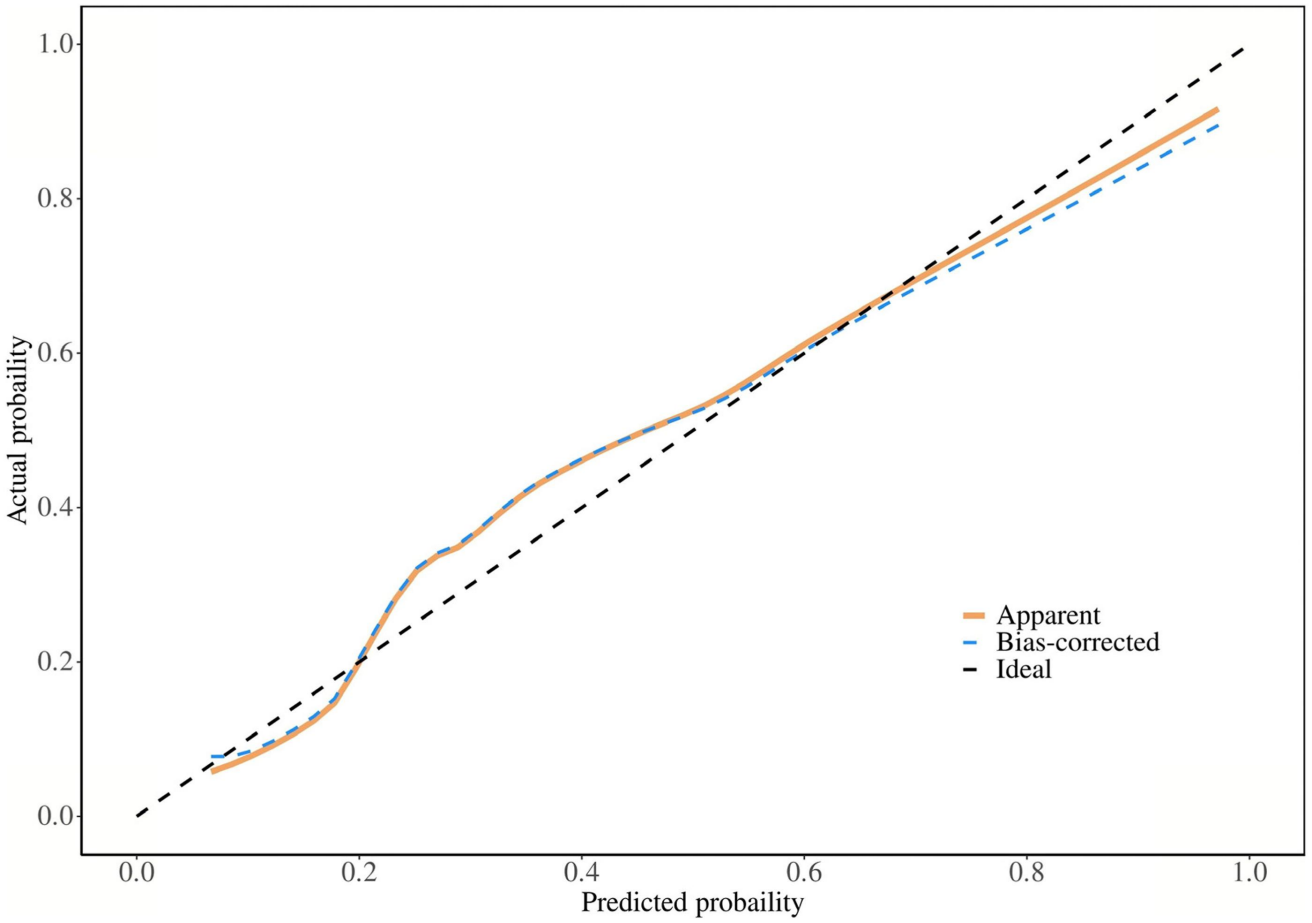
Calibration curve for Model 3.

**Figure 3:**
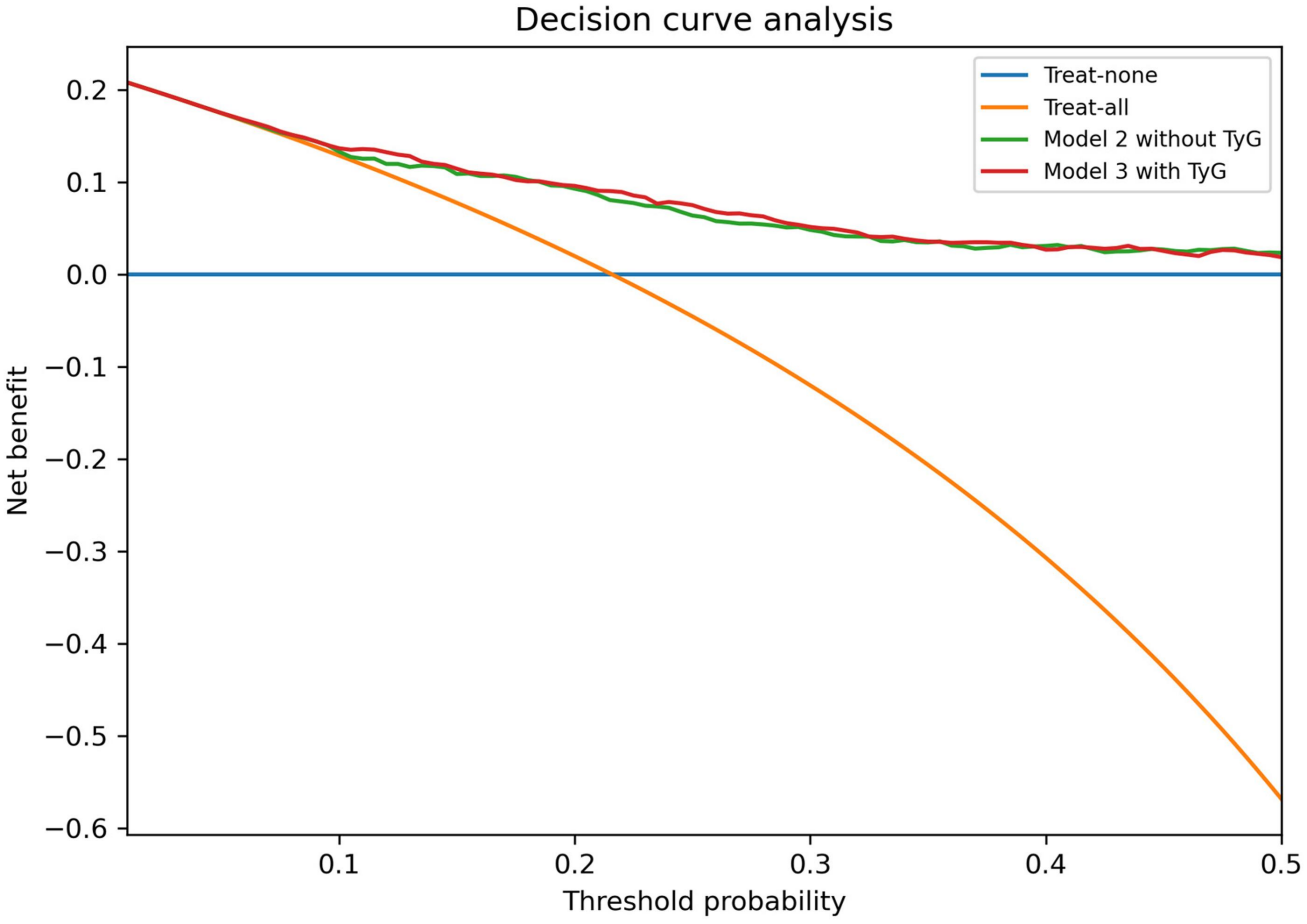
Decision curve analysis (DCA) showing the clinical utility of model 3.

To control for potential confounders, hierarchical multivariable logistic regression models were constructed. Model 1 (Base Model), which forced the inclusion of clinical variables such as age, sex, BMI, LVEF, and diabetes mellitus, showed that only history of alcohol consumption (OR = 2.41), peak cTnT (OR = 1.02), and peak NT-proBNP (OR = 1.01) were significantly associated with MACE (all *p* < 0.05) (Table 4). The predictive performance of this model, measured by the area under the ROC curve (AUC), was 0.72 (Figure 1a). Model 2 (Parsimonious Model) retained variables that were significant in the univariable analysis (*p* < 0.05) for the multivariable analysis. The results showed that peak cTnT (OR = 1.02) and peak NT-proBNP (OR = 1.01) remained independent predictors of MACE (both *p* < 0.001) (Table 5). The AUC of this model improved to 0.75 (Figure 1b). Model 3 (Core Model) added TyG index groups (using T1 as the reference) to Model 2. After adjusting for covariates including age, sex, BMI, LVEF, diabetes mellitus, cTnT, and NT-proBNP, the T2 group (medium TyG) was independently associated with a significantly increased risk of in-hospital MACE, with an OR 2.78 times that of the T1 group (low TyG) (OR = 2.78, 95% CI: 1.38–5.59, *p* = 0.004) (Table 6). In contrast, no statistically significant difference in MACE risk was observed for the T3 group (high TyG) compared to the T1 group (*p* > 0.05). The core model (Model 3) gave the best predictive performance, with its AUC increasing to 0.79 (95% CI: 0.75–0.83) (Figure 1c). A comprehensive evaluation of Model 3 performance showed: (a) Excellent discrimination (AUC = 0.79); (b) Good calibration, with the calibration curve indicating high consistency between predicted and observed probabilities (Figure 2), and a non-significant Hosmer-Lemeshow test result (*p* = 0.883), confirming a good model fit; c) High clinical utility, as decision curve analysis (DCA) demonstrated that using this model for clinical decision-making provided significant net clinical benefit across a wide range of threshold probabilities compared to default strategies (treat-all or treat-none) (Figure 3). To further evaluate the incremental value of the TyG stratification on top of existing predictors, we compared Model 3 with Model 2 using NRI, IDI, and updated receiver operating characteristic (ROC) curve analysis. After incorporating TyG stratification, the AUC increased from 0.754 in Model 2 to 0.769 in Model 3, with a *Δ*AUC of 0.015 (95% CI: 0.000–0.031; DeLong *P* = 0.049). The classification NRI resulting from TyG stratification was 0.075 (95% CI: 0.000–0.145; *P* = 0.042), the continuous NRI was 0.266 (95% CI: 0.133–0.402; *P* < 0.001), and the IDI was 0.008 (95% CI: 0.002–0.014; *P* = 0.015) (Table 7). These results suggest that incorporating TyG stratification improved both the model's risk reclassification ability and its discriminant slope. In the updated decision curve analysis, Model 3 demonstrated higher net benefit than Model 2 and the default strategy within the clinically relevant threshold range. At a preset threshold probability of 20%, the net benefits for Model 3, Model 2, treat-all, and treat-none were 0.096, 0.093, 0.020, and 0, respectively (Table 8).

**Table 4:** Multivariate logistic regression analysis for in-hospital MACE (model 1).

Variables	β	S.E.	Z value	*P* value	OR (95% CI)
Intercept	−2.01	0.32	−6.36	<0.001	0.13 (0.07–0.25)
Drinking					
No					1.00 (Reference)
Yes	0.88	0.39	2.24	0.025	2.41 (1.12–5.20)
Cardiac troponin T, pg/mL	0.02	0.01	2.25	0.024	1.02 (1.01–1.04)
NT-proBNP, pg/mL	0.01	0	4.41	<0.001	1.01 (1.01–1.01)

Odds ratios (ORs) and 95% confidence intervals (CIs) were calculated using multivariate logistic regression analysis. Model 1 was adjusted for variables showing statistical significance in univariate analysis.

OR, odds ratio; CI, confidence interval; NT-proBNP, N-terminal pro-B-type natriuretic peptide; MACE, major adverse cardiovascular events.

**Table 5:** Multivariate logistic regression analysis for in-hospital MACE (model 2).

Variables	β	S.E.	*Z* value	*P* value	OR (95% CI)
Intercept	−2.04	0.2	−10.45	<0.001	0.13 (0.09–0.19)
Cardiac troponin T, pg/mL	0.02	0.01	3.51	<0.001	1.02 (1.01–1.04)
NT-proBNP, pg/mL	0.01	0	6.87	<0.001	1.01 (1.01–1.01)

dds ratios (ORs) and 95% confidence intervals (CIs) were obtained using multivariate logistic regression analysis. Model 2 represents a streamlined model including the most statistically significant predictors.

OR, odds ratio; CI, confidence interval; NT-proBNP, N-terminal pro-B-type natriuretic peptide; MACE, major adverse cardiovascular events.

**Table 6:** Multivariate logistic regression analysis for in-hospital MACE (model 3).

Variables	β	S.E.	Z value	*P* value	OR (95% CI)
Intercept	−2.58	0.32	−8.08	<0.001	0.08 (0.04–0.14)
Triglyceride–glucose tertiles					
Tertile 1					1.00 (Reference)
Tertile 2	1.02	0.36	2.87	0.004	2.78 (1.38–5.59)
Tertile 3	0.28	0.38	0.73	0.463	1.33 (0.63–2.81)
Cardiac troponin T, pg/mL	0.02	0.01	3.68	<0.001	1.02 (1.01–1.04)
NT-proBNP, pg/mL	0.01	0	6.74	<0.001	1.01 (1.01–1.01)

Odds ratios (ORs) and 95% confidence intervals (CIs) were estimated using multivariate logistic regression analysis. Model 3 represents the core model incorporating triglyceride–glucose (TyG) tertiles to assess their independent association with in-hospital MACE.

OR, odds ratio; CI, confidence interval; TyG, triglyceride–glucose index; NT-proBNP, N-terminal pro-B-type natriuretic peptide; MACE, major adverse cardiovascular events.

**Table 7:** Incremental predictive value of TyG stratification beyond model 2.

Metric	Model2	Model3	Improvement	95%CI	*P* value
AUC	0.754	0.769	0.015	0.000–0.031	0.049
Categorical NRI	–	–	0.075	0.000–0.145	0.042
Event NRI	–	–	0.043	−0.017–0.107	0.168
Non-event NRI	–	–	0.031	−0.006–0.067	0.089
Continuous	–	—	0.266	0.133–0.402	<0.001
IDI	–	–	0.008	0.002–0.014	0.015

**Table 8:** Net benefit at selected threshold probabilities.

Threshold probability	Treat-none	Treat-all	Model2	Model3	Model3-Model2
15%	0.000	0.078	0.109	0.115	0.006
20%	0.000	0.020	0.093	0.096	0.003
25%	0.000	−0.045	0.064	0.075	0.011
30%	0.000	−0.120	0.048	0.052	0.003

#### 3.2.3 Interaction analysis between TyG tertiles and medication Use

No significant multiplicative interactions were detected in the core model between TyG index tertiles and the use of ACEI/ARB/ARNI (*P* for interaction = 0.315) or beta-blockers (*P* for interaction = 0.076) on the risk of in-hospital MACE (Table 9). This indicates that the J-shaped association between the TyG index and short-term prognosis was consistent regardless of background therapy with these agents.

**Table 9:** Interaction analysis between TyG tertiles and medication Use for in-hospital MACE.

Variables	TyG Tertile 1	TyG Tertile 2	TyG Tertile 3	*P* for interaction
ACEI/ARB/ARNI Use				0.315
Non-User, OR (95% CI)	1 [Reference]	2.52 (1.18–5.38	1.18 (0.55–2.53)	
User, OR (95% CI)	0.89 (0.52–1.52)	1.38 (0.84–2.27)	0.98 (0.61–1.58)	
β-blocker Use				0.076
Non-User, OR (95% CI)	1 [Reference]	1.60 (0.67–3.83)	0.46 (0.17–1.25)	
User, OR (95% CI)	1.06 (0.66–1.70)	1.58 (1.04–2.40)	1.19 (0.80–1.79)	

TyG, triglyceride-glucose index; OR, odds ratio; CI, confidence interval.

#### 3.2.4 Nomogram visualization based on the core model

**Figure 4:**
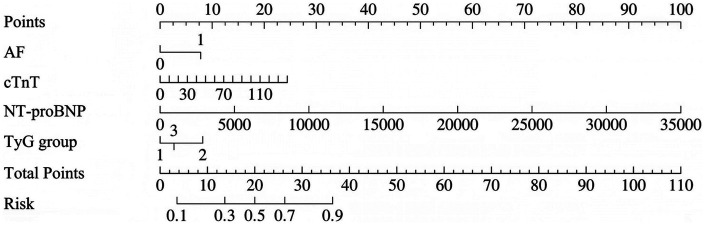
Nomogram based on the final regression coefficients of model 3 for predicting in-hospital MACE risk. To use: locate the patient's values on each predictor axis (AF, TyG group, peak cTnT, peak NT-proBNP), draw vertical lines to the points axis, sum the four points, then project the total points vertically to the bottom risk probabilit*y* axis to read the predicted probability.

To facilitate the clinical application of the core model (Model 3), a nomogram was constructed based on its final regression coefficients (Figure 4). This tool integrates four key predictors: atrial fibrillation (AF) (0 = no, 1 = yes), TyG index category (1 = T1, 2 = T2, 3 = T3), peak cTnT level, and peak NT-proBNP level. Users can locate the corresponding scores for an individual patient on the respective axes of the nomogram, sum these scores to obtain a total points value, and directly read the corresponding predicted probability of in-hospital MACE occurrence on the bottom risk probabilit*y* axis. A higher total score indicates the patient's greater risk of in-hospital MACE.

### 3.3 Nonlinear relationship between the TyG index and in-hospital MACE

**Figure 5:**
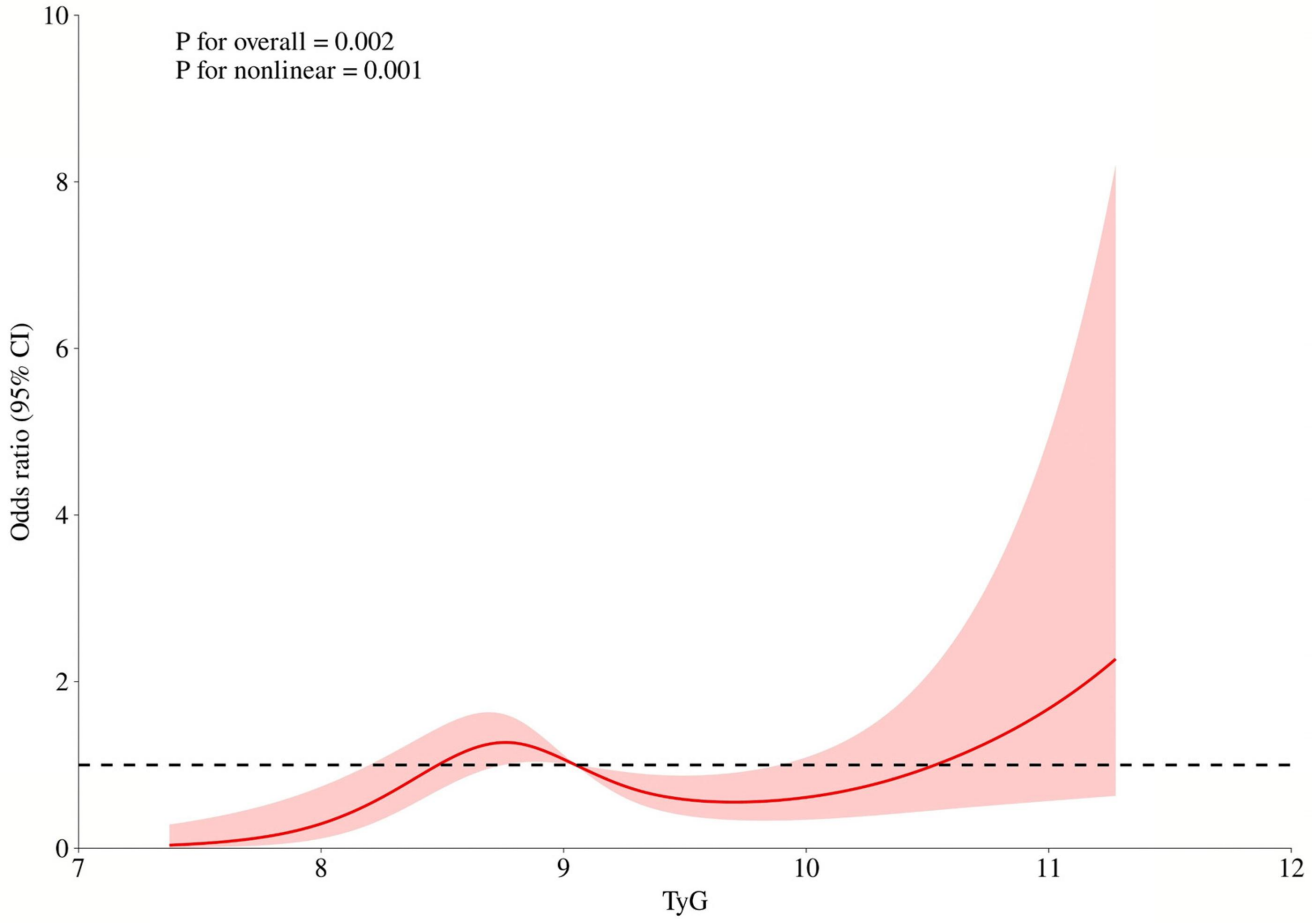
Restricted cubic spline fit of the relationship between TyG index and odds ratio of in-hospital MACE. The red shaded area indicates the 95% confidence intervals.

The relationship between the TyG index (as a continuous variable) and the risk of in-hospital MACE was explored using restricted cubic splines (RCS). The likelihood ratio test indicated a significant nonlinear association (*p* for overall association = 0.002, *p* for nonlinearity = 0.001) (Figure 5). Figure 5 displays the restricted cubic spline fit for the relationship between the TyG index and the odds ratio of in-hospital MACE (with the red shaded area indicating the 95% confidence intervals). It shows a distinct J-shaped curve: the MACE risk remained relatively stable when the TyG index was below a certain threshold, but increased sharply once this threshold was exceeded. The inflection point identified by the RCS analysis was located at TyG = 9.05. Piecewise logistic regression was performed to quantify the risk change on either side of this inflection point. In the range where the TyG index was <9.05, each 1-unit increase in the TyG index was not associated with a statistically significant change in MACE risk (OR = 1.04, 95% CI: 0.62–1.77, *P* = 0.875). Conversely, in the range where the TyG index was ≥9.05, each 1-unit increase in the TyG index was associated with a 124% significant increase in the risk of MACE (OR = 2.24, 95% CI: 1.26–3.96, *P* = 0.006) (Table 10).

**Table 10:** Threshold effect analysis of the TyG Index on in-hospital MACE.

Variables	β	S.E.	*Z* value	*P* value	Adjusted OR (95% CI)
Overall effect					
TyG index	0.012	–	–	–	1.45 (1.09–1.94)
Two-piecewise logistic regression model					
Inflection point	9.05				
TyG < 9.05	0.04	0.27	0.16	0.875	1.04 (0.62–1.77)
TyG ≥ 9.05	0.81	0.29	2.77	0.006	2.24 (1.26–3.96)

A two-piecewise logistic regression model was applied to examine the potential threshold effect of the triglyceride–glucose (TyG) index on in-hospital MACE risk. The inflection point was determined using a recursive algorithm.

OR, odds ratio; CI, confidence interval; TyG, triglyceride–glucose index; MACE, major adverse cardiovascular events.

### 3.4 Subgroup analysis

**Figure 6:**
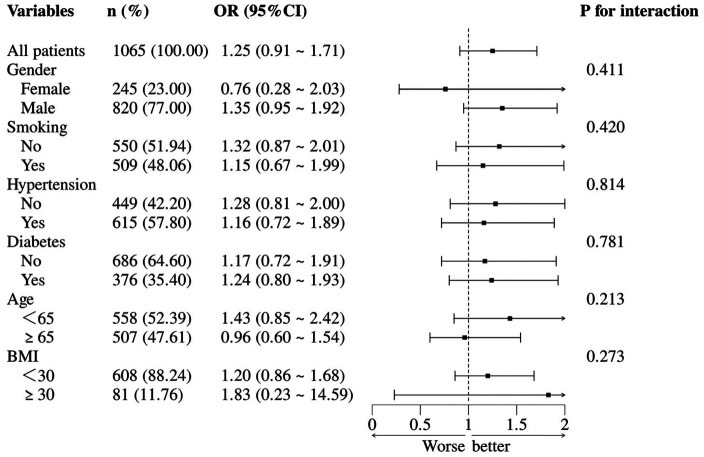
Forest plot of the subgroup analysis for the correlation between TyG index and in-hospital MACE.

**Figure 7:**
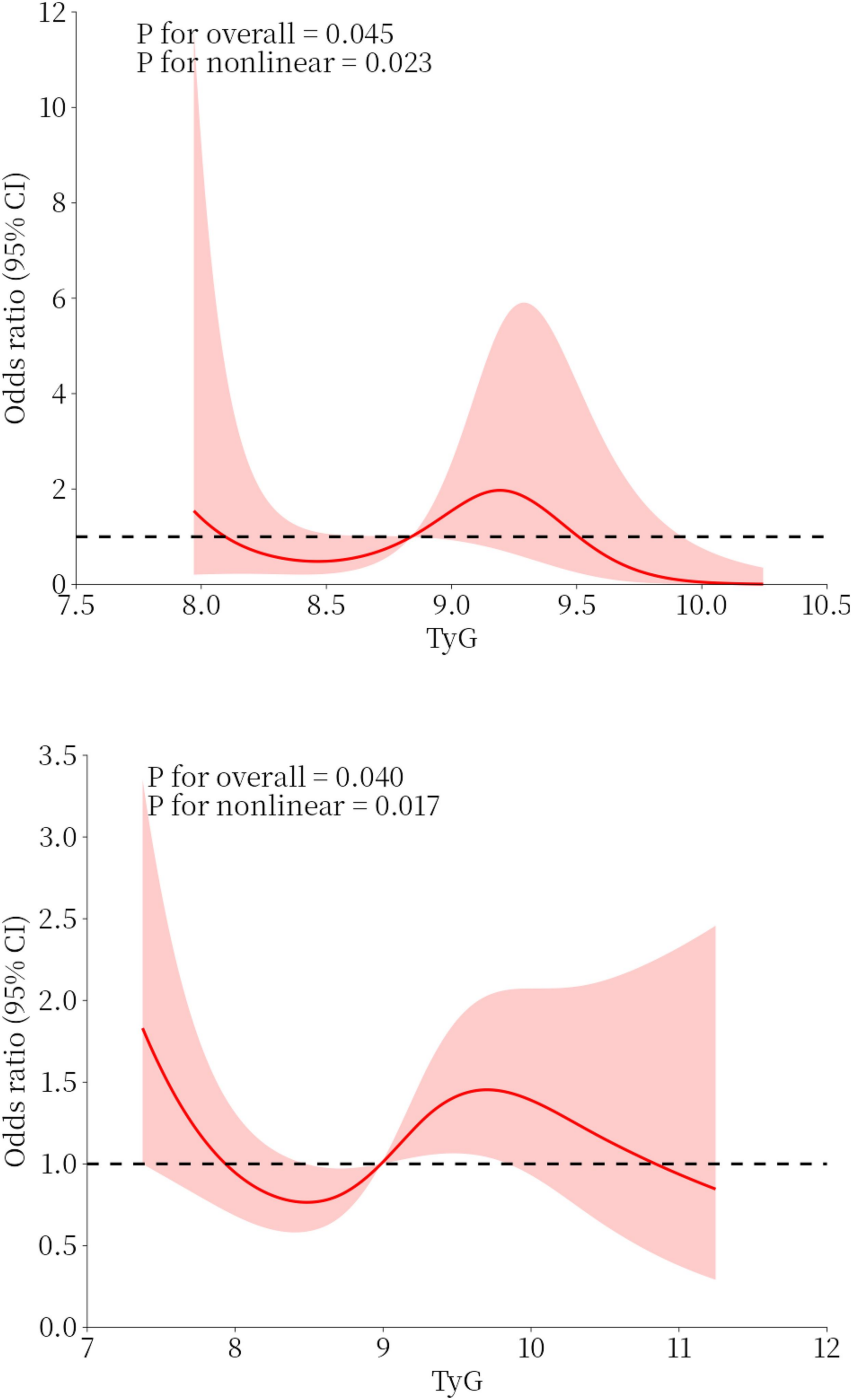
**(a)** Sensitivity analysis in non-diabetic subgroup. **(b)** Sensitivity analysis excluding patients with cardiogenic shock.

To assess the consistency of the association between the TyG index (stratified by the inflection point: ≥9.05 vs. <9.05) and the risk of in-hospital MACE across different clinical subgroups, we performed stratified multivariable logistic regression analyses, adjusting for covariates from the core model (Model 3), by sex, smoking status, history of hypertension, history of diabetes mellitus, age (<65 years/≥65 years), and BMI (<30 kg/m^2^/ ≥ 30 kg/m^2^). Analysis of the entire cohort, after stratification by the inflection point, indicated that the TyG index (≥9.05 vs. <9.05) was not significantly associated with MACE risk (OR = 1.25, 95% CI: 0.91–1.71, *p* = 0.169). More importantly, the *p* -values for interaction were greater than 0.05 in all subgroup analyses (Figure 6), indicating no significant heterogeneity in the association between the TyG index (stratified by the inflection point) and in-hospital MACE risk across patient subgroups defined by sex, smoking status, hypertension, diabetes, age, and BMI (All *p* for interaction > 0.05). These findings further support the existence of a consistent J-shaped association pattern between the TyG index and the risk of in-hospital MACE. Furthermore, to specifically evaluate whether the observed J-shaped association was driven by diabetic patients, a sensitivity analysis restricted to the non-diabetic subgroup (*n* = 689, 64.6%) was performed. Restricted cubic spline analysis within this subgroup confirmed that the J-shaped association between the continuous TyG index and in-hospital MACE risk remained consistent (Figure 7a). This finding reinforces that the nonlinear relationship is a more universal characteristic in the acute myocardial infarction population, independent of diabetes status.

### 3.5 Sensitivity analysis excluding patients with cardiogenic shock on admission

After excluding 32 patients who presented with cardiogenic shock on admission, the baseline characteristics and core findings remained materially unchanged (detailed data not shown but available upon request). Crucially, the J-shaped nonlinear association between the continuous TyG index and in-hospital MACE persisted (*p* for overall association = 0.040, *p* for nonlinearity = 0.017). The recalculated inflection point was 8.98, approximating the original value of 9.05 (Figure 6B). In the core multivariable logistic regression model (Model 3)(Table 11) applied to this truncated cohort, the T2 (medium TyG) group continued to exhibit the highest odds of in-hospital MACE compared to the T1 (low TyG) group (adjusted OR = 2.72, 95% CI: 1.35–5.48, *p* = 0.005). This sensitivity analysis confirms that the observed J-shaped relationship between the TyG index and in-hospital MACE is robust and not confounded by the acute metabolic disturbances of pre-existing cardiogenic shock.

**Table 11:** Multivariable logistic regression analysis for in-hospital MACE (model 3) after excluding patients with cardiogenic shock on admission.

Variables	β	S.E.	*Z* value	*P* value	OR (95% CI)
Intercept	−2.59	0.32	−8.09	<0.001	0.08 (0.04–0.14)
Triglyceride–glucose tertiles					
Tertile 1					1.00 (Reference)
Tertile 2	1.00	0.36	2.80	0.005	2.72 (1.35–5.48)
Tertile 3	0.30	0.38	0.78	0.434	1.35 (0.64–2.85)
Cardiac troponin T, pg/mL	0.02	0.01	3.73	<0.001	1.02 (1.01–1.04)
NT-proBNP, pg/mL	0.01	0	6.73	<0.001	1.01 (1.01–1.01)

Odds ratios (ORs) and 95% confidence intervals (CIs) were estimated using multivariate logistic regression analysis. Model 3 represents the core model incorporating triglyceride–glucose (TyG) tertiles to assess their independent association with in-hospital MACE.

OR, odds ratio; CI, confidence interval; TyG, triglyceride–glucose index; NT-proBNP, N-terminal pro-B-type natriuretic peptide; MACE, major adverse cardiovascular events.

## 4 Discussion

This large-scale retrospective cohort study of 1,065 patients with AMI provides the first systematic evidence of a significant J-shaped nonlinear association between the Triglyceride-glucose (TyG) index and the risk of in-hospital Major Adverse Cardiovascular Events (MACE), with an identified inflection point at TyG = 9.05. A landmark finding was that patients with a medium TyG level (T2 group) faced the highest risk of in-hospital MACE, and this remained significantly elevated even after extensive multivariable adjustment [adjusted OR = 2.78 compared to the low TyG (T1) group]. This pattern challenges the conventional paradigm of a simple linear positive correlation between the TyG index and cardiovascular risk. Furthermore, incorporating TyG index stratification significantly enhanced the predictive performance for in-hospital MACE (AUC = 0.79), with the model demonstrating good calibration and clinical utility. Subgroup analyses further confirmed the consistency of this J-shaped association across patients with diverse clinical characteristics.

To further elucidate the heterogeneous prognostic implications of different MACE components and identify whether the overall MACE risk was driven by a single dominant adverse event, we performed a secondary analysis of the individual components of the composite MACE endpoint across TyG index tertiles, with the full incidence data presented in Table 1. Our analysis revealed that the overall MACE risk associated with TyG index tertiles was not driven by a single dominant component, but rather by the combined effect of multiple adverse cardiovascular events. Malignant arrhythmia was the most frequent MACE component across all TyG groups, with an incidence of 9.9% in T1, 13.6% in T2, and 14.5% in T3, showing a stepwise increasing trend that aligned with the overall MACE risk gradient. Notably, recurrent myocardial infarction was the only individual MACE component that demonstrated a statistically significant difference across the three TyG tertiles (*P* = 0.0238), with an incidence peak in the T2 group (3.3%) that closely mirrored the J-shaped association of the composite MACE endpoint. No significant between-group differences were observed in the incidence of all-cause death, acute heart failure, or cardiogenic shock across the TyG index tertiles (all *p* > 0.05), although these events contributed to the overall composite MACE event count in our cohort. These component-specific observations reinforce the notion that the TyG index influences a spectrum of adverse events rather than a single phenotype, providing a more granular foundation for interpreting the overarching J-shaped relationship.

### 4.1 Interpretation of key findings and comparison with previous studies

The core contribution of this study lies in elucidating the nonlinear relationship between the TyG index and short-term prognosis in AMI patients. We observed a distinct J-shaped pattern: after an initial slight decline, the MACE risk remained relatively stable when the TyG index was below 9.05; however, once this threshold was exceeded, the risk increased sharply with each unit increment in the TyG index (OR = 2.24 per 1-unit increase, representing the average slope of the ascending limb in the TyG ≥ 9.05 range). This J-shaped association starkly contrasts the linear positive relationships frequently reported in previous studies involving broader cardiovascular populations or longer-term follow-ups (16, 17). This discrepancy may stem from our specific focus on the acute phase of AMI—a unique pathophysiological window. During this period, intense stress responses, sympathetic overactivation, and metabolic disturbances are markedly amplified, potentially causing the relationship between the TyG index and outcomes to manifest a more complex pattern distinct from that observed in stable conditions or long-term follow-up. Notably, while the risk was highest in the medium TyG (T2) group, the elevated OR for TyG ≥  9.05 also indicates that patients with very high TyG levels remain at considerable risk, underscoring that both moderate and severe insulin resistance states confer increased vulnerability during the acute infarct period.

More importantly, the finding that the medium TyG group (T2) had the highest MACE risk warrants in-depth exploration. It suggests that during the acute phase of AMI, the highest risk is not necessarily associated with the most severe degree of insulin resistance (IR). Instead, a moderate IR state might harbor a unique pathophysiological vulnerability. In this context, it is worth emphasizing that the TyG index has been well validated as a reliable surrogate for IR. Although we could not perform a direct head-to-head comparison with the homeostasis model assessment of insulin resistance (HOMA-IR) because of the absence of routine fasting insulin measurements in our retrospective dataset, previous studies in patients undergoing percutaneous coronary intervention have demonstrated that the TyG index correlates well with HOMA-IR and may even outperform it as a prognostic marker (18, 19). This provides a solid theoretical basis for our use of the TyG index as the primary IR metric and supports its applicability in the acute AMI setting. Our findings resonate with trends suggesting nonlinear associations of the TyG index observed in other populations by Hao et al. (20) and Tao et al. (21). However, this study is the first to explicitly confirm and precisely quantify this J-shaped association, including its inflection point, specifically in an AMI population during the critical in-hospital period. Moreover, both the prespecified subgroup analysis and the sensitivity analysis in non-diabetic patients demonstrated that this J-shaped relationship persisted irrespective of diabetes status, indicating that it is not merely driven by established diabetes but likely reflects a broader metabolic vulnerability inherent to the acute myocardial infarction state.

Further reinforcing the robustness of this nonlinear association, the sensitivity analysis excluding 32 patients who presented with cardiogenic shock on admission yielded materially unchanged results. The J-shaped curve was preserved (*p* for nonlinearity = 0.017), the recalculated inflection point (TyG = 8.98) closely approximated the original value of 9.05, and the T2 group remained independently associated with the highest risk of in-hospital MACE (adjusted OR = 2.72, 95% CI: 1.35–5.48, *p* = 0.005). These convergent findings effectively address the concern that the observed association might be an artifact of reverse causality driven by the acute metabolic derangements of cardiogenic shock. Instead, they demonstrate that the J-shaped relationship is a stable, intrinsic characteristic of the acute myocardial infarction population, independent of extreme hemodynamic compromise at presentation.

### 4.2 Potential mechanisms underlying the J-shaped association

As a reliable surrogate marker of IR, the association between the TyG index and cardiovascular risk is rooted in the broad pathophysiological processes mediated by IR (22). However, the J-shaped association revealed by our study, particularly the phenomenon of the highest risk at medium TyG levels, suggests the existence of unique mechanisms during the acute phase of AMI that extend beyond the severity of IR alone.

**Figure 8:**
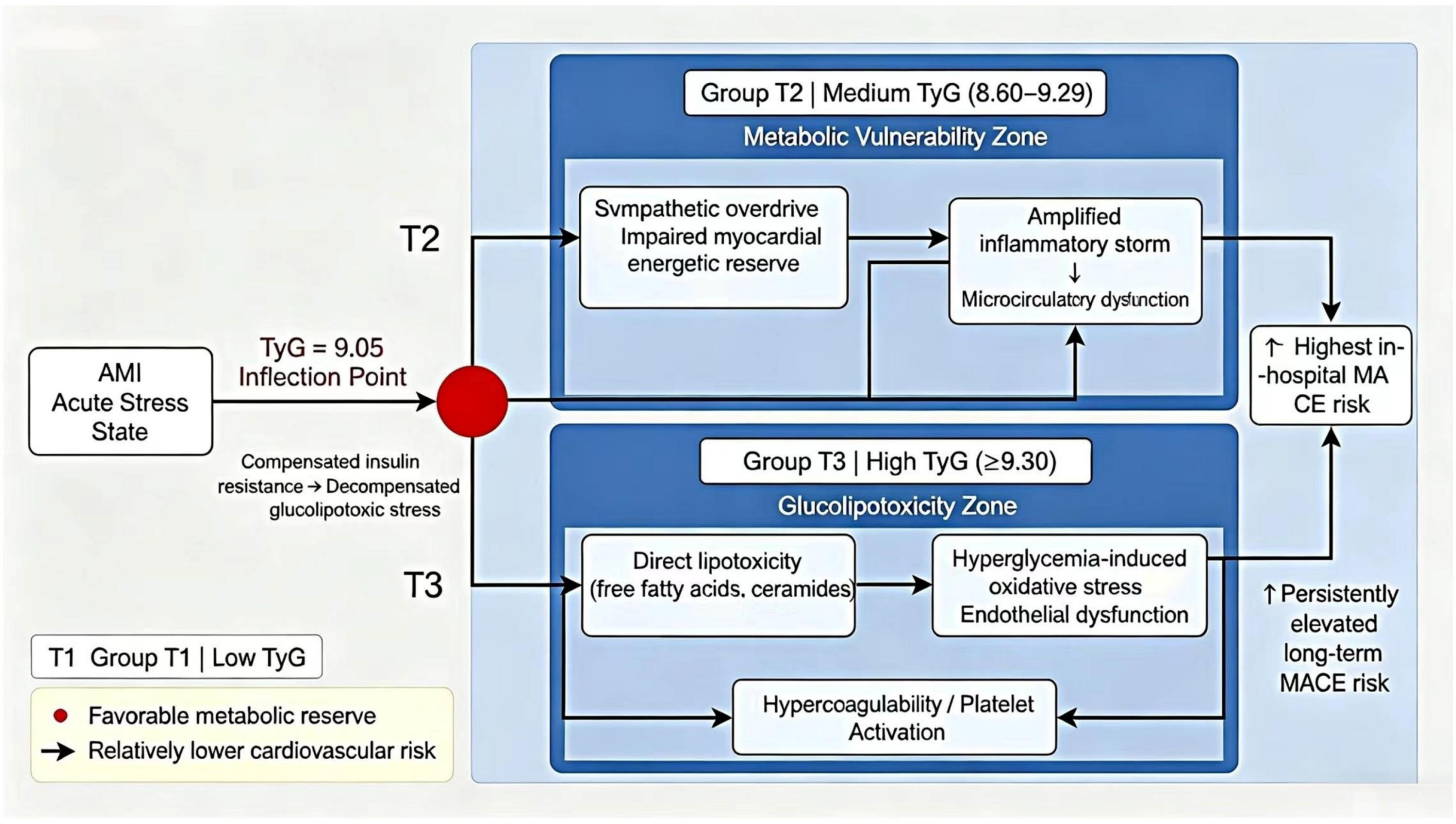
Conceptual mapping of distinct pathophysiological pathways in the metabolic vulnerability zone (T2) vs. glucolipotoxic zone (T3) along the TyG index continuum.

(a) The “Metabolic Vulnerability” Hypothesis: Baseline characteristics indicated that patients in the T2 group already exhibited clear features of metabolic dysregulation (e.g., higher BMI, FPG, TG, and lower HDL-C), yet the degree of their metabolic abnormality was less extreme than in the T3 group. We hypothesize that this “subclinical” or “moderate” IR state, when confronted with the major insult of AMI, may possess a metabolic reserve insufficient to cope with the acute stress. This could lead to an imbalance in energy metabolism, exacerbating the inflammatory storm, endothelial dysfunction, and impaired myocardial repair (23), ultimately manifesting as the highest event risk. In addition to the vulnerability inherent in the moderate IR state, acute metabolic lability may further amplify risk in this subgroup. An AMI triggers a massive catecholamine surge and systemic inflammatory response that can profoundly destabilize metabolic homeostasis. It is conceivable that patients with moderate chronic IR (T2) possess a fragile equilibrium that is easily disrupted, manifesting as pronounced fluctuations in glucose and free fatty acids—fluctuations that independently provoke oxidative stress, endothelial dysfunction, and electrophysiological instability. In contrast, individuals with a stably high TyG (T3) may have developed partial metabolic adaptation to a chronic glucolipotoxic milieu, thus exhibiting a less labile profile during the acute insult. This differential metabolic volatility within the T2 group represents a promising explanatory pathway for the J-shaped curve that merits direct evaluation in future studies. In contrast, patients in the T1 group are more likely to have a relatively intact metabolic reserve, while those in the T3 group, despite more severe IR, might have partially activated or desensitized specific pathological pathways resulting from chronic exposure to a glucolipotoxic environment. They may also have received more intensive metabolic management. Furthermore, in the moderate IR state, potential overactivation of the sympathetic nervous system and catecholamine release could exacerbate myocardial oxygen consumption and microcirculatory dysfunction (24), thereby increasing event risk. (b)Pathophysiological Significance of the Inflection Point: The restricted cubic spline analysis revealed a statistically derived inflection point at TyG = 9.05, above which the risk of in-hospital MACE escalated sharply. To enhance the biological plausibility of this threshold, we sought correspondence with validated metrics of insulin resistance. Although fasting insulin was not measured in our cohort and thus HOMA-IR could not be directly calculated, the inflection point TyG = 9.05 aligns closely with cut-offs demonstrated to signify the presence of insulin resistance. Notably, in a recent prospective analysis of the Atherosclerosis Risk in Communities (ARIC) cohort involving 12,543 middle-aged and older adults without diabetes, Xing et al. identified optimal TyG index cut-offs for predicting incident type 2 diabetes of 8.8–8.9 in men and 8.6–8.9 in women, which corresponded well with HOMA-IR thresholds of 2.8–3.2 and 2.4–3.2, respectively (25). This finding directly supports that a TyG index above approximately 9.0 represents a state of established insulin resistance. Furthermore, several investigations in patients with coronary artery disease have reported that TyG indices around 9.0–9.1 serve as discriminative thresholds for both prevalent metabolic syndrome and subsequent major adverse cardiovascular events (21, 26). Therefore, the inflection point at TyG = 9.05 plausibly represents a biological watershed: below this level, the metabolic milieu may still compensate for the acute ischemic stress through preserved substrate flexibility and neurohormonal regulation; above it, coexisting glucolipotoxicity, endothelial dysfunction, and amplified platelet reactivity create a self-reinforcing cycle that sharply elevates the risk of in-hospital MACE. Consistent with this notion, once the TyG index exceeds 9.05, significant insulin resistance coupled with overt dyslipidemia and dysglycemia may trigger synergistic amplification of glucolipotoxicity. Hyperglycemia can directly impair endothelial function, promoting oxidative stress and thrombus formation (27–29). Furthermore, this metabolic milieu is associated with increased basal vascular smooth muscle tone and reduced sensitivity to vasodilators such as nitric oxide, which can compromise coronary microvascular function and impair myocardial perfusion—a critical determinant of outcomes in AMI (30, 31).Concurrently, elevated free fatty acids and their toxic metabolites (e.g., ceramides) can induce cardiomyocyte apoptosis and mitochondrial dysfunction (32). Against the backdrop of unstable hemodynamics and a highly activated sympathetic state following AMI, this synergistic glucolipotoxic effect can readily precipitate or exacerbate heart failure, malignant arrhythmias, and cardiogenic shock. Recent studies also suggest that enhanced platelet activity and coagulation abnormalities associated with a TyG index above the inflection point may contribute (33, 34), further increasing the risk of thrombotic events. As illustrated in Figure 8, the J-shaped relationship can be understood as a switch from a state of metabolic vulnerability (T2) to uncompensated glucolipotoxicity (T3). In the T2 zone around the inflection point, inadequate metabolic reserve combined with sympathetic hyperactivity is thought to precipitate catastrophic acute events despite only moderate insulin resistance. Conversely, in the T3 zone, although chronic exposure to high glucose and lipids may have triggered some adaptive or desensitized responses, the direct toxic effects on the myocardium, endothelium, and coagulation system become dominant, explaining the steep rise in risk beyond TyG ≥ 9.05. Embedding the TyG index into this framework helps to translate the statistical inflection point into a clinically meaningful biological construct. This empirical observation—that the T2 and T3 groups did not differ significantly with respect to established surrogates of infarct severity (peak cTnT and CK-MB), cardiac function (LVEF), or the prevalence of multivessel disease and cardiogenic shock-further reinforces the interpretation that the J-shaped relationship is unlikely to be an artifact driven by a systematic imbalance in conventional clinical prognosticators between these two groups. It suggests instead that the risk gradient across the TyG continuum is primarily attributable to the intrinsic metabolic milieu rather than to disparities in infarct size or hemodynamic severity. Future prospective studies with standardized angiographic core laboratory analyses and systematic electrocardiographic adjudication are warranted to further delineate the interplay between metabolic indices and procedural determinants of AMI prognosis. (c)Potential Influence of Neurohormonal Factors and Treatment Response: The baseline differences in medication usage, with the highest rates of ACEI/ARB/ARNI and beta-blocker therapy observed in the high TyG (T3) group, likely reflect physicians' responses to the higher prevalence of comorbidities like diabetes and hypertension rather than being drivers of the elevated risk in the T2 group. Importantly, a formal interaction test confirmed that the association between TyG tertiles and in-hospital MACE was not modified by these medications (all *p* for interaction > 0.05). Furthermore, the T2 and T3 groups did not significantly differ in established prognostic markers of infarct severity, such as peak cTnT, LVEF, or the prevalence of cardiogenic shock. These findings collectively argue against the “confounding by indication” hypothesis-that T2 patients were simply a clinically “sicker” cohort—and instead reinforce the concept of a unique metabolic vulnerability intrinsic to the moderate insulin resistance state.

### 4.3 Clinical implications and translational value

Our findings carry significant implications for clinical practice: (a) Refinement of Risk Stratification: the results strongly suggest that risk assessment for AMI patients, particularly in the acute phase, must move beyond the simplistic linear assumption of “higher TyG equals higher risk” and instead account for its nonlinear and non-monotonic effect. Clinicians should be especially vigilant regarding the patient subgroup with medium TyG levels (specifically within the second tertile, approximately 8.60–9.29)-a potentially underestimated high-risk population-as well as paying heightened attention to patients whose TyG index exceeds the inflection point of 9.05. (b) Optimization of Predictive Tools: The core predictive model integrating TyG index stratification, cTnT, and NT-proBNP provided superior discriminatory ability compared to traditional models. The nomogram derived from this model offers a practical tool for rapid, bedside, individualized risk assessment, facilitating more precise allocation of monitoring resources and early intervention. (c) Novel Perspectives for Intervention Strategies: These findings indicate that differentiated management strategies may be warranted for patients with different TyG levels. For patients with medium TyG levels, intensified hemodynamic and rhythm monitoring during the acute phase is advisable. Should monitoring reveal signs of instability, timely interventions-such as adjustment of vasoactive medications, diuretics for incipient heart failure, or antiarrhythmic therapy-could be triggered. For patients with a TyG index ≥ 9.05, strategies involving more aggressive glycemic control, which have demonstrated cardiovascular benefits, should be considered to mitigate the short-term high risk of glucolipotoxicity. These include, (avoiding fluctuations, potentially with insulin infusion protocols), triglyceride management (e.g., intensification of statin therapy or use of fibrates), and therapies to improve insulin sensitivity (considering the potential role of agents like SGLT2 inhibitors or GLP-1 receptor agonists. This tailored approach aims to translate risk stratification into actionable clinical pathways. (d) Incremental Prognostic Value and Clinical Utility Beyond Traditional Predictors: In addition to the discrimination performance reflected by AUC, we further supplemented Net Reclassification Improvement (NRI) and Integrated Discrimination Improvement (IDI) analyses as well as targeted decision curve analysis (DCA) to verify the incremental prognostic value and clinical utility of TyG index stratification beyond conventional risk predictors. Compared with Model 2 (the parsimonious model), Model 3 (the core model with TyG tertile stratification) showed a significantly improved AUC from 0.754 to 0.769 (*Δ*AUC = 0.015, 95% CI: 0.000–0.031, DeLong *P* = 0.049). Meanwhile, the addition of TyG stratification yielded a categorical NRI of 0.075 (95% CI: 0.000–0.145, *P* = 0.042), a continuous NRI of 0.266 (95% CI: 0.133–0.402, *P* < 0.001), and an IDI of 0.008 (95% CI: 0.002–0.014, *P* = 0.015). These objective quantitative indicators confirm that TyG index stratification provides independent and incremental value in both risk reclassification and overall discriminative ability beyond conventional prognostic markers. Furthermore, the updated DCA was performed with a clinically actionable intervention defined as escalation to intensive in-hospital monitoring for high-risk patients. Given the overall in-hospital MACE incidence of 21.7% in the present cohort, a threshold probability of 20% was used as the clinically reasonable cutoff. At this threshold, the net benefit of Model 3 (0.096) was higher than that of Model 2 (0.093), and both models were substantially superior to the treat-all and treat-none strategies. These results overcome the inherent limitation of AUC in reflecting incremental predictive value and provide robust evidence supporting the integration of TyG index stratification into routine risk assessment for AMI patients. By identifying high-risk individuals more accurately, this refined risk stratification may facilitate timely escalation of monitoring, early metabolic intervention, and targeted prophylaxis against recurrent ischemia and malignant arrhythmias, thereby further improving the short-term prognosis of AMI patients during the high-risk in-hospital stage.

### 4.4 Strengths and limitations

The strengths of this study include its large sample size, adherence to strict diagnostic criteria for AMI, the objective identification of a nonlinear relationship through multivariable adjustments and restricted cubic spline (RCS) analysis, a comprehensive evaluation of model performance, and supportive evidence for the association's consistency from subgroup analyses.

However, several limitations should be acknowledged. First, the single-center, retrospective design precludes the establishment of causality, although extensive multivariable adjustments were performed to minimize confounding. Moreover, owing to the retrospective nature, certain procedure-related details-including time from symptom onset to reperfusion, precise culprit lesion localization, angiographic collateral flow grading, and imaging indices of microvascular obstruction-were not systematically documented and could not be incorporated into the multivariable models. We cannot completely exclude the possibility of residual confounding from these unmeasured factors. Second, this study focused solely on short-term in-hospital outcomes; the impact of the TyG index on long-term prognosis warrants further investigation. Third, despite TyG being a well-validated surrogate marker for insulin resistance, this study did not directly compare it against the HOMA-IR due to the absence of routine fasting insulin measurements in our retrospective dataset. Given evidence that TyG may outperform HOMA-IR in certain PCI patient populations (18, 19), future prospective studies are warranted to verify whether TyG provides superior incremental prognostic value over HOMA-IR in the acute AMI setting. Fourth, our study did not collect data on socioeconomic status, detailed dietary habits, physical activity levels, or medication adherence history. These factors may independently affect glucose and lipid metabolism (thus influencing the TyG index) as well as cardiovascular prognosis in patients with AMI. Therefore, these unmeasured factors may represent potential residual confounding in the present analysis. Fifth, and most relevant to the J-shaped association, in-hospital metabolic variability could not be assessed. The TyG index was based on a single admission fasting sample. The hypothesis that a moderate baseline TyG combined with acute glycemic/lipid lability may be more detrimental than a stably high TyG provides a plausible explanation for the excess T2 risk. However, our retrospective data lacked serial glucose and lipid profiles, precluding calculation of standard deviation, coefficient of variation, or time in range. We therefore cannot distinguish the prognostic contributions of baseline insulin resistance from those of acute metabolic fluctuations. Whether the J-shaped relationship reflects intrinsic vulnerability or acts as a surrogate for unmeasured acute-phase instability remains unresolved. Future prospective studies with continuous glucose monitoring and serial lipid profiling are needed. Finally, as with any observational study, residual confounding from unmeasured factors cannot be entirely ruled out.

## 5 Conclusion

In conclusion, this study demonstrates a significant J-shaped nonlinear association between the TyG index and the risk of in-hospital MACE in patients with AMI, with an identified inflection point at TyG = 9.05. Crucially, patients with moderate TyG levels (within the T2 tertile, TyG ≈ 8.60–9.29) faced a risk comparable to that observed in patients with the highest TyG levels (e.g., >11). This finding challenges the conventional linear and monotonic perception of TyG-associated risk. It underscores the necessity of considering the nonlinear and non-monotonic effect of the TyG index in the acute-phase risk stratification of AMI patients, identifying the moderate TyG population as a high-risk subgroup requiring particular attention. Prospective, multicenter studies are warranted to validate this association and to explore subsequent individualized intervention strategies for improving short-term prognosis in AMI.

## Data Availability

The original contributions presented in the study are included in the article/Supplementary Material, further inquiries can be directed to the corresponding author/s.
